# Challenges in cerebrospinal fluid shunting in patients with glioblastoma

**DOI:** 10.1186/s12987-018-0101-x

**Published:** 2018-06-04

**Authors:** Bujung Hong, Manolis Polemikos, Hans E. Heissler, Christian Hartmann, Makoto Nakamura, Joachim K. Krauss

**Affiliations:** 1 Department of Neurosurgery, Hannover Medical School, Hannover, Germany; 2 Institute for Pathology, Department for Neuropathology, Hannover Medical School, Hannover, Germany; 3 Department of Neurosurgery, Cologne Mehrheim Medical Center, University of Witten/Herdecke, Cologne, Germany

**Keywords:** Glioblastoma, Ventriculoperitoneal shunt, Hydrocephalus, Cerebrospinal fluid

## Abstract

**Background:**

Cerebrospinal fluid (CSF) circulation disturbances may occur during the course of disease in patients with glioblastoma. Ventriculoperitoneal shunting has generally been recommended to improve symptoms in glioblastoma patients. Shunt implantation for patients with glioblastoma, however, presents as a complex situation and produces different problems to shunting in other contexts. Information on complications of shunting glioma patients has rarely been the subject of investigation. In this retrospective study, we analysed restropectively the course and outcome of glioblastoma-related CSF circulation disturbances after shunt management in a consecutive series of patients within a period of over a decade.

**Methods:**

Thirty of 723 patients with histopathologically-confirmed glioblastoma diagnosed from 2002 to 2016 at the Department of Neurosurgery, Hannover Medical School, underwent shunting for CSF circulation disorders. Treatment history of glioblastoma and all procedures associated with shunt implementation were analyzed. Data on follow-up, time to progression and survival rates were obtained by review of hospital charts and supplemented by phone interviews with the patients, their relations or the primary physicians.

**Results:**

Mean age at the time of diagnosis of glioblastoma was 43 years. Five types of CSF circulation disturbances were identified: obstructive hydrocephalus (n = 9), communicating hydrocephalus (n = 15), external hydrocephalus (n = 3), trapped lateral ventricle (n = 1), and expanding fluid collection in the resection cavity (n = 2). All patients showed clinical deterioration. Procedures for CSF diversion were ventriculoperitoneal shunt (n = 21), subduroperitoneal shunt (n = 3), and cystoperitoneal shunt (n = 2). In patients with lower Karnofsky Performance Score (KPS) (< 60), there was a significant improvement of median KPS after shunt implantation (p = 0.019). Shunt revision was necessary in 9 patients (single revision, n = 6; multiple revisions, n = 3) due to catheter obstruction, catheter dislocation, valve defect, and infection. Twenty-eight patients died due to disease progression during a median follow-up time of 88 months. The median overall survival time after diagnosis of glioblastoma was 10.18 months.

**Conclusions:**

CSF shunting in glioblastoma patients encounters more challenge and is associated with increased risk of complications, but these can be usually managed by revision surgeries. CSF shunting improves neurological function temporarily, enhances quality of life in most patients although it is not known if survival rate is improved.

## Background

Glioblastoma still has a dismal prognosis: significantly limiting quality of life and survival of patients suffering from this aggressive tumor [1, 2]. Maximal safe surgical resection followed by adjuvant combined radiochemotherapy is one of the most important factors to improve overall survival of patients [3–5]. Cerebrospinal fluid (CSF) circulation disturbances may occur during the course of disease and reduce a patient’s quality of life as well as treatment capability significantly. Neurological deterioration associated with the development of hydrocephalus has been observed in 5–10% of patients with glioblastoma [6–13].

Ventriculoperitoneal shunting has generally been recommended to improve symptoms in glioblastoma patients with CSF circulation disturbances [6–13]. Shunt implantation in patients with glioblastoma, however, presents as a complex situation and produces different problems to shunting in other contexts [14, 15]. Although hydrocephalus is seen in a significant number of patients with glioblastoma, there are only a few studies which have concentrated on outcome of CSF shunting in this fragile group of patients [6, 8–13, 27]. Even more so, information on complications of shunting rarely has been the subject of investigation [11–13, 54].

Against this background, we sought to analyse the course and outcome of glioblastoma-related CSF circulation disturbances after shunt management in a consecutive series of patients within a period of over a decade.

## Methods

For this retrospective study a data base of 723 glioblastoma patients with histopathologically-confirmed glioblastoma diagnosed from 2002 to 2016 at the Department of Neurosurgery, Hannover Medical School, was reviewed. All patients that underwent shunting for CSF circulation disorders were included. Treatment history of glioblastoma, including surgical interventions and postoperative therapy were reviewed. In addition, all procedures associated with shunt implementation, including type of shunt, technical problems, and complications, were analysed. Data on follow-up, time to progression and survival rates were obtained by review of hospital charts and supplemented by phone interviews with the patients, their relations or the primary physicians.

Radiological evaluation of glioblastoma and tumor progression, in general, was made using contrast-enhanced MRI. CSF circulation disturbance was defined as disproportionate enlargement of inner and/or outer CSF spaces within the cranial vault or CSF collections in the resection cavity in postoperative imaging studies associated with the appearance of new clinical symptoms. Follow-up MRIs were obtained regularly at 3 months intervals. After CSF shunting, all patients had X-ray shuntograms confirming valve settings and the location of catheters.

Microsurgical resection for tumor removal was performed according to standard surgical techniques for tumors not involving eloquent areas of the brain. Depending on tumor localization, neuronavigation, electrophysiological mapping, and/or 5-aminolevulinic acid (5-ALA) were applied intraoperatively [16, 17]. Gross total microsurgical resection was achieved when the neurosurgeon determined that all areas of visible tumor were resected intraoperatively. Resection was defined as subtotal when remnants of tumor were left behind.

Shunt surgery was performed after diagnosis of a clinically-relevant CSF circulation disturbance. Depending on the cause of the CSF circulation disturbance, ventriculoperitoneal, subduroperitoneal, or cystoperitoneal CSF diversion was performed. Programmable valve systems were implanted in 28 patients. While only four patients had a programmable valve (Codman & Shurtleff, Inc. Raynham, USA), in 24 patients, this was combined with implantation of a gravitational anti-siphon device [18]. Two patients received a medium pressure CSF-flow control valve system (Medtronic, Minneapolis, MN, USA). Due to poor accessibility of the ventricle system, a neuronavigation system was used for insertion of the ventricular catheter in three patients [16, 17].

### Statistical methods

Sigma Stat software (version 3.5; Systat Software, Inc. California, USA) was used for statistical analysis. To analyse the differences between groups, Student’s *t* test was used. The survival rate was estimated by the Kaplan–Meier method. Summary data were presented as median. Statistical significance was defined as a probability value less than 0.05. Measures were presented as mean ± standard deviations.

## Results

### Patient characteristics

Overall, 30/723 patients with histopathologically-proven glioblastoma (4.2%) underwent CSF shunting procedures during the study period. Patient characteristics are presented in Table 1. Mean age at the time of diagnosis of glioblastoma was 43 years (range 1 to 79 years). Initial surgical treatment included stereotactic biopsy (n = 7), partial resection (n = 6), subtotal resection (n = 8), and complete resection (n = 9). Two of 7 patients, who had stereotactic biopsy, underwent subsequent surgical resection after confirmation of the histopathological diagnosis of glioblastoma, since tumor location was considered accessible. In 18 patients the infiltrated ependymal wall of the ventricle system was opened during surgical resection.

**Table 1:** Demographics and clinical characteristics of glioma patient group

Variable	n (%)
Sex	
Male	20 (66.7)
Female	10 (33.3)
Age at diagnosis	
< 60	23 (76.7)
≥ 60	7 (23.3)
Number of microsurgical tumor resections prior to shunt implantation	
None	5 (16.7)
1 time	17 (56.7)
2 times	3 (10.0)
3 times	4 (13.3)
4 times	1 (3.3)
Type of CSF circulation disturbance	
Obstructive hydrocephalus	9 (30.0)
Communicating hydrocephalus	15 (50.0)
External hydrocephalus	3 (10.0)
Trapped ventricle	1 (3.3)
Expanding CSF collection in resection cavity	2 (6.7)
Type of CSF diversion	
Ventriculoperitoneal	21 (70.0)
Subduroperitoneal	3 (10.0)
Cystoperitoneal	2 (6.7)
Combined two catheters	
Frontal horn + temporal horn	2 (6.7)
Frontal horn bilateral	1 (3.3)
Expanding cyst + temporal horn	1 (3.3)

*CSF* cerebrospinal fluid

### Treatment

After histopathological confirmation of glioblastoma, temozolomide (TMZ) was administered in 14 patients concurrently with radiotherapy according to current standard therapy [19]. One patient received ACNU/VM26 within the NOA-1 protocol [20]. Three children were treated in accordance to HIT-GBM (German Society of Paediatric Oncology and Haematology) treatment protocols. Eighteen patients received conventional-fractionated partial brain radiotherapy with a total dose of 54–60 Gy (single dose, 1.8–2.0 Gy) starting within 6 weeks after initial resection. Eight patients underwent one or more microsurgical resections for recurrent GBM, of whom three patients tumor resection once, 5 patients twice, and 1 patient 3 times.

### CSF circulation problems

**Fig. 1:**
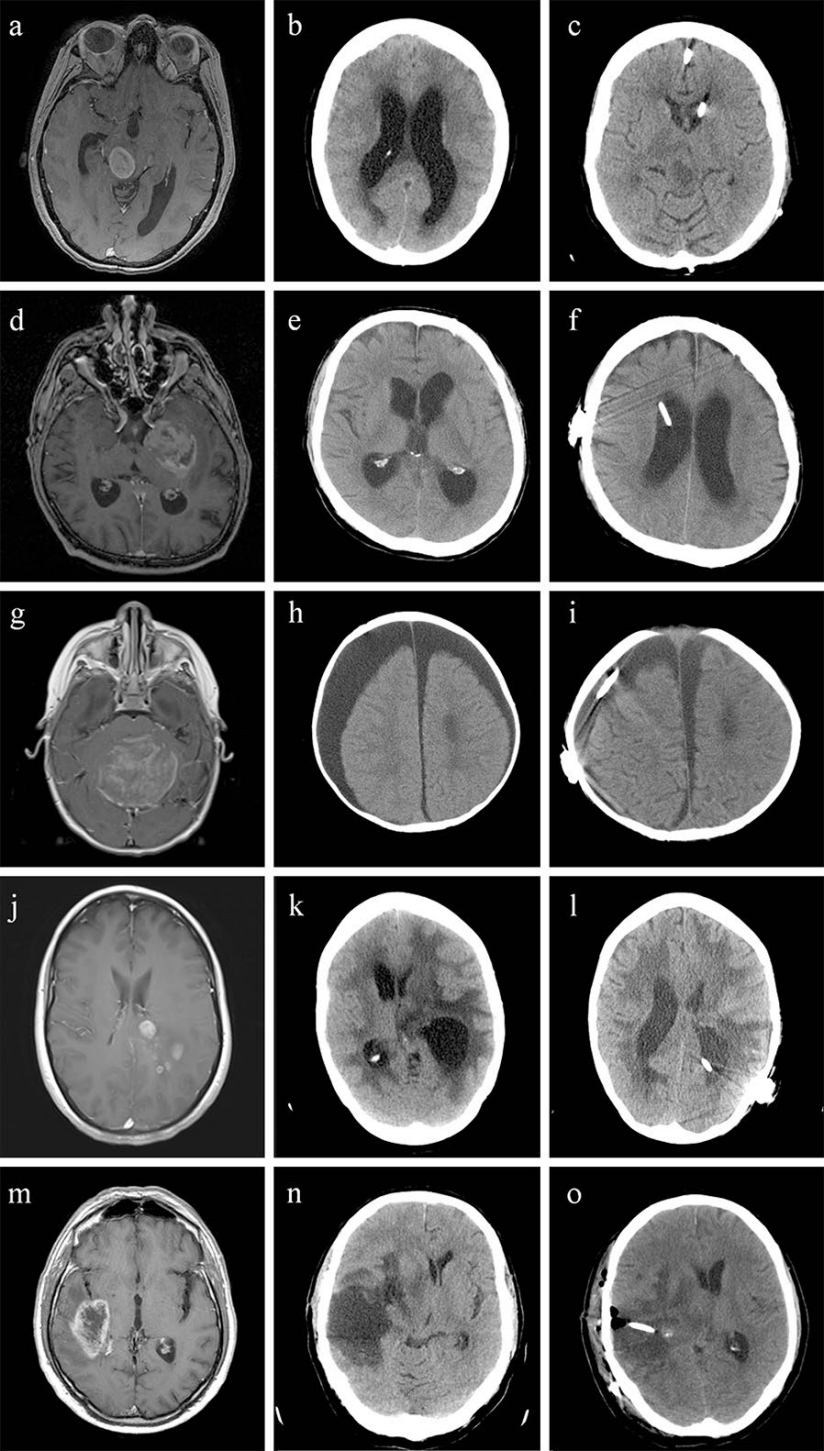
Images of glioblastoma patients: left column T1-weighted MR image after administration of gadolinium, centre collumn native CT scan showing CSF circulation disturbances and right collumn postoperative native CT scan after shunt placement. **a** A 41-year-old woman shows a pontomesencephalic glioblastoma in with compression of the aqueduct. **b** Widening of the lateral ventricles due to obstructive hydrocephalus. **c** Reduction of ventricular size after implantation of a ventriculoperitoneal shunt. **d** A 64-year-old man shows a glioblastoma in the left temporomesial lobe. **e** Ventricular enlargement due to communicating hydrocephalus. **f** The intracranial catheter in situ after implantation of a ventriculoperitoneal shunt. **g** A 1-year-old boy shows a midline glioblastoma, which resulted initially in obstructive hydrocephalus. **h** External hydrocephalus with extensive subdural hygroma. **i** After implantation of a subduroperitoneal shunt. **j** A 43-year-old woman shows a glioblastoma in the left parietal lobe/subcortical white matter. **k** Three weeks after tumor resection, CT imaging reveals isolated extension of the left posterior horn with local compression of adjacent structures and midline shift. **l** A shunt catheter in the posterior horn after implantation of a ventriculoperitoneal shunt. **m** A 69-year-old man shows a glioblastoma in the left temporal lobe. **n** A space occupying fluid collection in the resection cavity. **o** The proximal shunt catheter inserted in the resection

Overall, five types of CSF circulation disturbances were identified: obstructive hydrocephalus (n = 9) (Fig. 1a), communicating hydrocephalus (n = 15) (Fig. 1d), external hydrocephalus (n = 3) (Fig. 1g), trapped lateral ventricle (n = 1) (Fig. 1j), and expanding CSF collection in the resection cavity (n = 2) (Fig. 1m). All patients showed clinical deterioration, mainly due to increase of intracranial pressure, predominantly presenting with headache, drowsiness, psychomotor slowing, hemiparesis, or aphasia.

### Treatment for CSF diversion

In five patients, a rapid symptomatic progression of hydrocephalus, as confirmed in the initial radiological images, was detected, so that a CSF shunt was implanted prior to surgical tumor resection. The other patients had one (n = 17) or repeated (n = 8) surgical tumor resection prior to shunt implantation. The type of CSF diversion included ventriculoperitoneal (n = 21), subduroperitoneal (n = 3), and cystoperitoneal shunting (n = 2). In four patients, two catheters were inserted in separate intracranial compartments due to combined causes of CSF circulation disturbances. Three patients who underwent subduroperitoneal shunting had previously undergone subdural drainage via a burr hole. Shunting was performed for persistent subdural CSF collection with impaired consciousness or focal neurological signs. Depending on the clinical and radiological findings, the programmable valve was initially set to 6–8 cm H_2_O (n = 24) for the ProGAV^®^ valve system and to 50–140 mm Hg (n = 4) for the Codman^®^ Hakim^®^ valve system. CSF sampling from 11 patients taken at shunt surgery showed no cytological evidence of tumor dissemination.

### Shunt failure and revision surgery

In five patients with programmable valve system, the valve pressure settings were subsequently adjusted due to over- or underdrainage as determined on clinical and radiological findings.

Shunt failure occurred in nine patients. The main symptoms of shunt failure were impairment of consciousness (n = 10), cephalgia (n = 8), and focal neurological deficits (n = 5). Other symptoms included seizures, gait disorder, and aphasia (n = 5). In three patients, routine follow-up imaging showed persistent hydrocephalus, and valve malfunction. Neither specific symptoms nor new neurological deficits were evident in these patients.

A total of 16 revision surgeries were performed in 9 (30.0%) adult patients (7 men, 2 women; mean age 55 years) due to various complications (Table 2), of which 11 (68.8%) revision surgeries were performed within the first year after shunt implantation. Overall, three patients (10%) required multiple revision surgeries (2 revision surgeries, n = 1; 3 revision surgeries, n = 1; 6 revision surgeries, n = 1). In three patients, the anti-siphon device was removed over the course of disease. The patient with 6 revision surgeries had delayed shunt malfunction, which occurred approximately 3 years after the time of implantation.

**Table 2:** Indications for revision surgery in nine patients with shunt malfunction on 16 occasions

Indication	n	Type of surgical revision
Wound dehiscence with pneumocephalus	1	Wound revision
Delayed fluid collection in resection cavity	1	Additional cystoperitoneal shunt implantation
Valve and proximal catheter obstruction	3	Catheter replacement
Proximal catheter obstruction	1	Valve and catheter replacement
Proximal catheter dislocation	1	Reinsertion
Distal catheter dislocation	1	Reinsertion
Valve malfunction	3	Valve replacement
Delayed trapped ventricle and CSF collection in cavity, valve malfunction	1	New implantation of proximal shunt catheters without anti-siphon device
Intracerebral abscess	1	Removal of ventriculoperitoneal shunt, implantation of external ventricle drainage
Delayed trapped ventricle and CSF collection in cavity	1	New implantation of proximal catheters
Persistent hydrocephalus despite adjustment of programmable valve	2	Removal of anti-siphon device

*CSF* cerebrospinal fluid

### Outcome

**Fig. 2:**
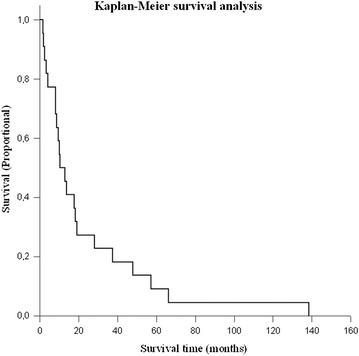
A Kaplan–Meier plot demonstrates overall survival in the cohort of 30 patients with glioblastoma who underwent shunt implantation due to CSF disturbances

Twenty-two patients temporarily benefitted from shunting with subsequent improvement of consciousness and neurological symptoms. Overall, the median KPS improved significantly from 50 to 70 after shunt implantation (p = 0.008 of which, six patients remained stable at a median KPS of 70 at 3 months postoperatively. The other two patients deteriorated further due to tumor progression within a median follow-up time of 5.5 months. When patients were dichotomized in two groups, there was a significant improvement of the median KPS after shunt implantation in those with a lower KPS (< 60) prior to shunt implantation (p = 0.019), while improvement in those with a higher prior KPS (≥ 60) did not reach statistical significance. The follow-up time of all patients ranged from 1 to 138 months with a median of 10 months. Twenty-eight patients died due to disease progression. Two patients were still alive at the time of writing manuscript. The median overall survival (mOS) time after diagnosis of glioblastoma was 10 months (Fig. 2). The two-year survival rate was 23.3%, 3-year survival rate 16.7%, and 5-year survival rate 6.7%.

## Discussion

Despite the development of hydrocephalus and the subsequent need for shunting in patients with glioblastoma is not an uncommon problem, this topic has attracted relatively little attention. Here we show that although shunting may not prolong overall survival, it significantly improves functional performance if only at least temporarily. The fact that there is relatively little information available on this issue most likely is due to circumstances which may have excluded these patients from larger studies or outcomes. Even less information is available on shunt complication during follow-up in this group of patients as summarized in Table 3.

**Table 3:** Reported studies of cerebrospinal fluid shunting in patients with glioblastoma

Author, year	Frequency, n (%)	Type of CSF circulation disturbance	Shunt complication
Marquardt et al. 2002 [7]	12/351 malignant gliomas (3.4)	CH (n = 12)	Multiple surgeries due to multiloculated hydrocephalus (n = 1)
Inamasu et al. 2003 [8]	5/50 GBM (10)	CH (n = 5)	None reported
Roth et al. 2008 [11]	16/530 GBM (3)	CH (n = 16)	Infection (n = 6), shunt malfunction (n = 1), overdrainage and hemorrhage (n = 1)
Montano et al. 2011 [6]	11/124 GBM (8.9)	CH (n = 7), OH (n = 2), fluid in resection cavity (n = 2)	None reported
de la Fuente et al. 2014 [12]	41/2433 gliomas WHO grade II–IV (1.7)	CH (n = 41)	Meningitis (n = 5), subdural hematoma (n = 5), haemorrhage (n = 1), infection (n = 6)
Fischer et al. 2014 [9]	11/151 GBM (7.3)	CH (n = 11)	n.a.
Esquanazi et al., 2017 [27]	20/57 thalamic GBM (35)	OH (n = 20)	n.a.
Behling et al. 2017 [10]	13/229 GBM (5.7)	IH (n = 11), EH (n = 2)	n.a.
Castro et al. 2017 [13]	64/841 GBM (7.6)	CH (n = 42), OH (n = 22)	Infection (n = 10), catheter occlusion (n = 1), combined overdrainage, ventriculitis and haemorrhage (n = 1)

*GBM* glioblastoma; *CH* communicating hydrocephalus; *OH* obstructive hydrocephalus; *IH* internal hydrocephalus; *EH* external hydrocephalus; *CSF* cerebrospinal fluid; *n.a.* not available

Among the aforementioned types of CSF circulation disturbances, communicating hydrocephalus appears to be the most frequent one. Communicating hydrocephalus may occur due to entry of blood into the CSF spaces during surgery, due to elevated CSF protein, secondary to radiotherapy-induced fibrosis of arachnoid granulations, or to leptomeningeal dissemination of tumor cells [6–9, 21–24]. All these events may finally result in obliteration of the subarachnoid spaces over the surface of the brain with reduction of CSF absorption. Some authors reported a significant correlation between ventricular opening during tumor resection and development of hydrocephalus [6, 9, 25], which, however, was not confirmed in other studies [10]. Multiple microsurgical resection for recurrent glioblastoma is likely to prolong survival [26]. However, it is also associated with an increased risk for communicating hydrocephalus [6]. Furthermore, previous radiotherapy increases the production of transforming growth factor-ß (TGF-ß) in cerebral tissues and glioma cells, supporting the transformation of fibroblasts into myofibroblasts, which promotes fibrosis of arachnoid granulations [21, 23].

The second most common type of CSF circulation disturbances in glioblastoma is obstructive hydrocephalus, which is caused by the obstruction of CSF pathways, mostly due to compression of the 3rd or the 4th ventricle, as typically detected in midline, cerebellar, or thalamic glioblastoma [27]. External hydrocephalus, trapped ventricle, and expanding space-occupying fluid collection in the resection cavity are seen more rarely. The pathological mechanism behind the development of external hydrocephalus is not fully understood. Some investigators have suggested that CSF absorption failure by widespread leptomeningeal and subependymal tumor metastases with simultaneous loss of ventricular compliance could be the underlying causes for the CSF collections in the subdural space [9, 28]. Others hypothesized that differential pressure between the ventricles and the subarachnoid spaces would allow CSF to pass from the ventricles to the subarachnoid space, and thus, result in CSF accumulation in the resection cavity or in the subdural space [29, 30]. Trapped ventricles occur typically as a complication of intraventricular hemorrhage [31–33]. Entry of blood in the ventricles during the surgery may result in adhesions and scarring of the ventricular wall, finally sealing off the posterior horn. CSF shunting is the treatment of choice to relieve the rapid ventricular dilatation.

Given the complexity of the development of CSF disturbances and the clinical condition, shunting in glioblastoma patients presents frequent challenges since treatment appears to be associated with increased risks for peri- and intraoperative complications. A higher incidence of shunt complications has been reported in the few studies focussing on this issue. Roth and colleagues have indicated that 8 of 16 glioblastoma patients with ventriculoperitoneal shunts had shunt-related complications, of which 3 patients died due to such complications [11]. De la Fuente analysed 62 patients with supratentorial glioma, of which 41 had glioblastoma. Among these patients, 27% had complications related to ventriculoperitoneal shunts [12]. Further, a more recent study reported that shunt complications required surgical revision in 4 of 12 (33%) high-grade glioma patients with ventriculo-/cystoperitoneal shunts [34]. In our study, shunt failure affected mainly the ventricular catheter and the valve system. Hence, proximal complications appear to be the major causes of shunt dysfunction, whereas abdominal complications do not appear to occur more frequently in glioblastoma. Shunt complications are manifest most frequently within the first year after implantation. Some authors have suggested elevation of CSF protein levels and dissemination of tumor cells to be the causes for proximal obstruction and valve defects [9, 34, 35]. The clinical symptoms of shunt failure in glioblastoma patients are not always readily recognizable, particularly in patients with a lower KPS, impaired consciousness, or pre-existing focal neurological deficits. Regular follow-up CT or MR scans might provide evidence for shunt failure. Cohen et al. [36], however, found no correlation between clinical symptoms and radiological findings of shunt failure.

Shift of anatomical landmarks as a result of perifocal edema, tumor growth, or distention of the resection cavity during the course of treatment can make the implantation of the proximal catheter technically more challenging in some instances [37–40] resulting in a higher risk for misplaced catheters [34]. In cases of poor accessibility to the ventricular system, neuronavigation is a valuable tool for achieving adequate placement of ventricular catheter [16, 17]. In cases of multiple CSF-filled compartments, insertion of separate catheters or even separate shunt systems is sometimes necessary. Such surgeries need to consider existing scars and previous craniotomies when draining the CSF compartments, thus making shunt surgery more challenging. Interestingly, there are hardly any studies in the literature dealing with the role of multiple intracranial catheters in patients with ventriculoperitoneal shunts.

Radiation and chemotherapy are prone to weaken the immune system [41]. Temozolomid chemotherapy which may induce lymphopenia and myelosuppression has been associated with poor immune surveillance leading to opportunistic infection in patients with malignant glioma [42, 43]. Other alkylating agents, such as Lomustine (CCNU) and PCV (Procarbazine—CCNU—Vincristine) induce predominantly neutropenia which can also increase the risk of infection [44]. Especially the combination of previous surgeries and radiochemotherapy can considerably contribute to wound-healing impairment. Furthermore, prolonged corticosteroid application, immobility, long hospitalisation and advanced age are other unfavorable factors often present in glioblastoma which increase the risk for infection [45, 46]. In some studies, up to 50% of glioblastoma patients with ventriculoperitoneal shunts experienced infection within 2 weeks after surgery [11]. In a recent study, Beez et al. reported 4 revision surgeries due to infection in 12 patients with high grade glioma and ventriculo-/cystoperitoneal shunts [34]. When shunt removal is inevitable, temporary external ventricle drainage is required. Shunt infection may be lethal in such patients [11].

Disease progress is associated with declined physical activity and some patients may be confined to bed for a long time. When patients are mobile, the integration of a gravity-assisted shunt valve can reduce the problem of siphoning and the occurrence of over drainage, and thus avoid posture-related headaches and the risk of subdural hygromas or hematoma [18, 47]. When patients are bedridden, however, gravity-assisted shunt valves should be avoided, since they may carry risk of underdrainage and persistent hydrocephalus [48]. A particular problem arises in such instances when patients are mobile initially but become bedridden in a later stage of the disease as detected in three patients of our series, making additional surgery to remove the gravity-assisted device unavoidable.

Although dissemination of glioblastoma within the ventricular system is well known [49], intraperitoneal metastasis via a ventriculoperitoneal shunt appears to occur very rarely [50–56]. We did not find any evidence of peritoneal seeding in any patient of our series. It has to be mentioned, however, that manifestation of glioblastoma in the abdominal cavity following ventriculoperitoneal shunting has been detected mainly by postmortem autopsy. Nevertheless, thus far, there is no systematic study using appropriate methods to detect metastatic seeding of glioblastoma cells into the peritoneal cavity via a shunt system intra vitam. Thus, the true incidence of intraperitoneal metastasis through shunting remains to be elucidated.

We acknowledge several limitations of our study, particularly related to its retrospective characters. We cannot determine the role of CSF shunt on survival time since the median KPS was quite low indicating a population with poorer prognosis. Furthermore, we did not study systematically the possibility of seeding of glioblastoma cells via the shunt system. In addition, it would have been be interesting to compare outcome between shunted and non-shunted GBM patients in a larger study.

## Conclusions

Different types of CSF circulation disturbances may occur during the course of disease in patients with glioblastoma. Shunting achieved temporary improvement for functional performance in the majority of the patients studied, but it is not known if shunting increases survival rate. Shunt implantation in patients with glioblastoma is more complex and burdened with a higher risk of complications, which, nevertheless, can usually be managed by revision surgery. Careful evaluation of the indication for CSF shunting and close postoperative follow-up is necessary.

## Abbreviations

5-ALA: 5-Aminolevulenic acid
CSF: cerebrospinal fluid
CT: computer tomography
GBM: glioblastoma
HIT-GBM: Hirntumor-Glioblastoma Multiforme
Gy: gray
KPS: Karnofsky Performance Score
mOS: median overall survival
MRI: magnetic resonance imaging
NOA: Neuroonkologische Arbeitsgemeinschaft
PCV: Procarbazine—CCNU—Vincristine
TGF-ß: transforming growth factor-ß
TMZ: temozolomide
